# Epidemic trends and characteristics of notifiable infectious diseases in mainland China during the COVID-19: a nationwide surveillance study, 2020–2024

**DOI:** 10.7189/jogh.16.04118

**Published:** 2026-04-10

**Authors:** Jingjuan Xu, Yang Xu, Zhenhua Dai, Zhenna Xu, Hui Cai, Zhiming Zhao, Wei Shang, Yanqi Dang

**Affiliations:** 1 Jinling Hospital, Affiliated Hospital of Medical School, Nanjing University, Nanjing, China; 2 Rizhao Center for Disease Control and Prevention, Rizhao, China; 3 Rizhao Sub-district Community Health Center, Rizhao, China; 4 Institute of Digestive Diseases, China-Canada Center of Research for Digestive Diseases, Longhua Hospital, Shanghai University of Traditional Chinese Medicine, Shanghai, China

## Abstract

**Background:**

The implementation of COVID-19 control and prevention measures has significantly influenced the incidence rates of multiple notifiable infectious diseases. We aimed to investigate the epidemiological trends of notifiable infectious diseases in mainland China from 2020 to 2024, a period spanning both stringent interventions and their subsequent relaxation.

**Methods:**

We systematically analysed surveillance data from the National Center for Disease Control and Prevention (2020–2024). We excluded COVID-19, monkeypox, and neonatal tetanus to ensure methodological consistency and comparability. We classified the diseases into Class A, B, and C notifiable infectious diseases and further grouped them by transmission routes: intestinal, respiratory, sexually transmitted and blood-borne, vector-borne/zoonotic, and others. We focused on incidence rates, mortality rates, seasonal patterns, and trends to inform future prevention and control strategies.

**Results:**

Between 2020 and 2024, mainland China recorded 38 notifiable infectious diseases (excluding COVID-19, monkeypox, and neonatal tetanus). The average incidence rate was 734.8945/100 000, showing an upward trend. Class A notifiable infectious diseases were extremely rare, Class B encompassed 25 types and showed a rising trend with minimal seasonal variation, and Class C included 11 types. Class C notifiable infectious diseases incidence remained relatively low from 2020 to 2022, but rose sharply in 2023 after the relaxation of COVID-19 restrictions, maintaining elevated levels in 2024, with pronounced winter/spring peaks observed, especially in 2023–2024. Respiratory infectious diseases (RIDs) exhibited the highest incidence, while blood-borne and sexually transmitted infectious diseases accounted for over 90.35% of the deaths.

**Conclusions:**

In mainland China, strict COVID-19 measures between 2020 and 2022 significantly reduced the incidence of RIDs. However, after COVID-19 management was downgraded and restrictions were relaxed in early 2023, these diseases resurged, demonstrating a ‘suppression-rebound’ effect.

Infectious diseases are illnesses caused by pathogenic microorganisms (including bacteria, viruses, and fungi) and parasites [1]. These diseases can spread extensively among humans and animals through various transmission pathways, including human-to-human, animal-to-human, and animal-to-animal transmission, thereby posing major public health risks [2]. China has implemented substantial initiatives to prevent and treat infectious diseases. The law governing this matter was originally enacted in 1989, underwent its first revision in 2004, and was further amended on 30 April 2025, with the revised version slated to come into effect on 1 September 2025. In China, managing public health emergencies has consistently stood as a paramount concern, receiving top-tier attention at the national level. Consequently, the country has developed and implemented a series of comprehensive emergency response plans. These plans underscore the critical role of early monitoring and warning systems, the protection of vulnerable populations, the disruption of transmission pathways, and the provision of medical treatment and support, all of which are essential strategies for the prevention and control of infectious diseases [3–5].

Nevertheless, between 1 and 31 May 2025, mainland China reported 1 077 760 cases of notifiable infectious diseases, resulting in 2043 deaths [6], indicating an ongoing public health threat. A fundamental aspect of controlling infectious diseases is the comprehensive analysis of their epidemiological trends and characteristics. The global patterns of infectious disease epidemics are shaped by a multitude of factors, including changes in prevention and control policies, advances in vaccination efforts, and shifts in social behaviours [7,8]. Numerous reports indicate that following the outbreak of COVID-19, initial containment measures effectively suppressed various infectious diseases; however, subsequent policy adjustments have led to resurgences or altered seasonal patterns of certain diseases [9–12]. To date, there is no comprehensive analysis of the COVID-19 influence on notifiable infectious diseases in mainland China.

To evaluate the influence of COVID-19 on these diseases, we used surveillance data from the National Notifiable Disease Reporting System spanning the years 2020 to 2024. We sought to examine trends and epidemiological features of infectious diseases to provide insights and evidence to improve disease prevention and control. We aimed to synthesise experiences and provide scientific evidence to address the emerging challenges of controlling and preventing infectious diseases.

## METHODS

### Data analysis

**Figure 1:**
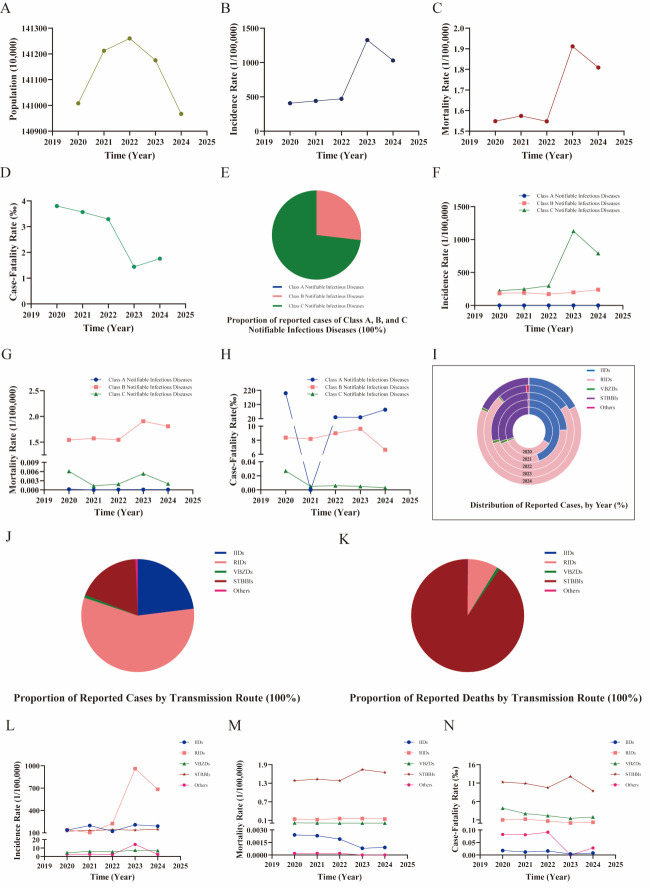
Comprehensive analysis of notifiable infectious diseases in Mainland China (2020–2024). **Panel A.** Total demographic and overall trends. **Panel B.** Demographic overall trends of incidence rate. **Panel C.** Demographic overall trends of mortality rate. **Panel D.** Demographic overall trends of the CFR of notifiable infectious diseases. **Panel E.** Proportion of reported cases of Class A, B, and C notifiable infectious diseases. **Panel F.** Trends in the incidence rate. **Panel G.** Trends in the mortality rate. **Panel H.** Trends in the CFR of Class A, B, and C notifiable infectious diseases. **Panel I.** Distribution of reported cases by year. **Panel J.** Proportion of reported cases by transmission route. **Panel K.** Proportion of reported deaths by transmission route. **Panel L.** Analysis of incidence rate based on transmission routes. **Panel M.** Analysis of mortality rate based on transmission routes. **Panel N.** Analysis of the CFR of notifiable infectious diseases based on transmission routes. CFR – case-fatality rate, IID – intestinal infectious disease, RID – respiratory infectious disease, STBBI – sexually transmitted and blood-borne infection, VBZD – vector-borne/zoonotic disease.

We obtained data on notifiable infectious diseases in mainland China (excluding Hong Kong, Macao, and Taiwan) between 2020 and 2024 from the ‘Notifiable Infectious Diseases Reports: Reported Cases and Deaths of National Notifiable Infectious Diseases’. The National Health Commission of the People's Republic of China published this report for the period from 1 January 2020 to 31 August 31 2022, and the Chinese Center for Disease Control and Prevention published it for the period from 1 September 2022 to 31 December 2024. We sourced the demographic data, excluding those for Hong Kong, Macao, and Taiwan, from the year-end total population figures published in the ‘China Statistical Yearbook 2024’ by the National Bureau of Statistics (**Figure 1**, Panel A). On 1 September 2022, the responsibility for disseminating infectious disease reports was transferred from the National Health Commission to the Center for Disease Control and Prevention. Owing to its integrated surveillance system and rigorous data quality controls, the system's standardised framework and protocols have ensured uniformity in case definitions, reporting requirements, statistical standards, and publication formats across the nation. Consequently, this has preserved data continuity and comparability, ensuring that analyses of epidemic trends remain unaffected. It is important to note that the data reflect reported cases, deaths, and incidence and mortality rates from the national surveillance system, which have been influenced by testing availability, healthcare-seeking behaviour, reporting adherence, and public health priorities during various phases of the COVID-19 pandemic. These factors may contribute to under-ascertainment, with the degree of under-ascertainment likely varying across different diseases and over time. We obtained data from publicly accessible, aggregated statistical reports that contained no personal identifiers.

### Exclusion of specific diseases

To maintain methodological consistency and ensure the validity of comparative analyses, we excluded COVID-19, monkeypox, and neonatal tetanus. COVID-19 was classified as a Class B notifiable disease on 20 January 2020, and was subject to Class A measures until 8 January 2023, when it shifted to standard Class B management. Due to its unprecedented scale and distinct impact on public health systems and intervention strategies, including COVID-19 data would disproportionately skew the analysis of trends in other infectious diseases. Consequently, we excluded it to facilitate a focused assessment of the effects of non-pharmaceutical interventions (NPIs) on infectious diseases other than COVID-19. Monkeypox was classified as a Class B notifiable infectious disease for management in China on 20 September 2023. Given that cases were negligible or non-existent prior to 2023, their inclusion would distort trend analyses and compromise data comparability across the study period (2020–2024). Furthermore, the incidence rate and mortality rates for neonatal tetanus are expressed per 1000 live births, reflecting a distinct methodology of data calculation and targeting a specific demographic. This unique denominator and specific demographic distribution create significant disparities when compared to other notifiable infectious diseases, thereby complicating the integration of neonatal tetanus data into comprehensive analyses and potentially introducing bias. Excluding neonatal tetanus ensures consistency in data units and facilitates more accurate cross-disease comparisons.

### Classification of notifiable infectious diseases

As of May 2025, the ‘Catalogue of Notifiable Infectious Diseases’ issued by the National Disease Control and Prevention Administration lists two Class A, 28 Class B, and 11 Class C notifiable infectious diseases in China. Excluding monkeypox, neonatal tetanus, and COVID-19, the remaining 38 diseases, classified based on their primary transmission routes, fall into the following five major categories: intestinal infectious diseases (IIDs), respiratory infectious diseases (RIDs), vector-borne/zoonotic diseases (VBZDs), sexually transmitted and blood-borne infections (STBBIs), and infectious diseases with other transmission routes [13]. Within this framework, viral hepatitis, including types A, B, C, D, E, and unspecified variants, is further categorised by the unique transmission pathways pertinent to each subtype (Table S1 in the **Online Supplementary Document**). It should be noted that the 2022 annual report for viral hepatitis subtypes (A, B, C, D, and E) used unverified monthly data and did not undergo the usual annual deduplication process. As a result, the reported totals were higher than they would have been after standard verification.

### Statistical analysis

We employed both descriptive epidemiological and descriptive statistical methods to examine the epidemiological characteristics and seasonal variations of notifiable infectious diseases in mainland China between 2020 and 2024. We evaluated the intensity and composition of disease using several metrics, including the incidence and mortality rates per 100 000 population, constituent ratio as a percentage, and case-fatality rate (CFR) per thousand. To determine the annual incidence and mortality rates, we used the officially reported data from the National Health Commission for the period from 2020 to August 2022, and from the Center for Disease Control and Prevention from September 2022 to 2024. We calculated these rates based on the total population at the end of the preceding year for each respective year. For 2022, in the absence of official annual reports, we estimated the rates using the 2021-year-end population, in accordance with the standard reporting methodology.

We performed seasonal analyses across various strata, including legal classifications (A, B, and C) and transmission route categories. It is important to acknowledge that seasonal trends observed at the aggregate class level may obscure significant and contrasting variations among individual diseases within the same classification. We calculated the monthly incidence rate using estimated mid-year population denominators. Given the unavailability of publicly accessible mid-year population data in China, we estimated mid-year populations for 2020–2024 by linear interpolation from the year-end population totals reported in the China Statistical Yearbook 2024 (Table S2 in the **Online Supplementary Document**). Specifically, we determined the mid-year population for a given year (y) as: Mid-year population_y_ = Year-end population_y −1_ – Year-end population_y_ / 2

We determined the 95% confidence intervals (CIs) for the monthly incidence rates using the Clopper-Pearson method, as the case counts are rare events in large populations. To quantify the seasonal patterns of disease incidence, we calculated the peak-to-trough ratio (PTR) and the seasonal index (SI) for each major disease category using monthly data from 2020 to 2024. We defined PTR as the ratio of the highest to the lowest monthly incidence rate within a calendar year, thereby reflecting the magnitude of seasonal fluctuations. We calculated SI using a moving-average method to assess the extent to which monthly incidence rates deviate from the annual average, with values >100 indicating peaks and values <100 indicating troughs.

Furthermore, we ranked notifiable infectious diseases by total reported cases and deaths, using absolute counts and proportions to reflect their impact on national surveillance and public health resources. To ensure that our conclusions were not skewed by a single dominant pathogen such as influenza, we conducted a sensitivity analysis. This involved recalculating the top five rankings without influenza to identify the leading diseases contributing to morbidity and mortality, independent of their significant yearly fluctuations.

We processed and analysed the national surveillance data using Microsoft Excel, 2021 (Microsoft Corporation, Redmond, Washington, USA), and did the visualisation using GraphPad Prism, version 10 (GraphPad Software, San Diego, California, USA)

## RESULTS

### Epidemiological profile of notifiable infectious diseases

During 2020–2024, mainland China reported 38 notifiable infectious diseases, excluding COVID-19, monkeypox, and neonatal tetanus, with all cases included in the analysis. There were 51 771 730 reported cases, with an average incidence rate of 734.8945/100 000, ranging from 407.4242 to 1326.8076/100 000. The incidence rate showed an overall increasing trend, peaking sharply in 2023, before declining slightly in 2024 (**Figure 1**, Panel B). There were 118 206 reported deaths, with an average mortality rate of 1.6782/100 000. The mortality rate remained relatively stable between 2020 and 2022, rose markedly in 2023, and then decreased slightly in 2024 while remaining at an elevated level (**Figure 1**, Panel C). The five-year CFR was 2.283‰. The overall trend from 2020 to 2024 was one of substantial decline, primarily driven by the surge in cases in 2023, followed by a modest rebound in 2024 that still left the CFR well below pre-2023 levels (**Figure 1**, Panel D; Table S3 in the **Online Supplementary Document**).

### Analysis of notifiable infectious diseases based on legal classification

Between 2020 and 2024, notifiable infectious diseases in mainland China showed a distribution of approximately 0.0002% for Class A, 26.8277% for Class B, and 73.1722% for Class C (**Figure 1**, Panel E). Class A notifiable infectious diseases included two types, totalling 100 cases and six deaths, with an average annual incidence of 0.0014/100 000 and a mortality rate of 0.0001/100 000. The low case numbers make these rates unstable and limit their interpretability. However, they highlight the success of prevention efforts, with an incidence rate below 0.003‰. Class B notifiable infectious diseases comprised 25 types during the study period, among which severe acute respiratory syndrome, poliomyelitis, and H7N9 had no reported cases. There were 13 889 142 reported cases, with an average incidence rate of 197.1588/100 000. Between 2020 and 2023, the reported incidence rate fluctuated between 172.1148 and 198.1371/100 000, before increasing to a peak of 238.956/100 000 in 2024. A total of 117 965 deaths occurred, with an average mortality rate of 1.6747/100 000. The reported mortality rate remained relatively stable from 2020 to 2022, ranging between 1.5422 and 1.5728/100 000. However, it increased markedly to 1.9062/100 000 in 2023 before declining slightly to 1.807/100 000 in 2024, which is still above the pre-2023 level. The CFR remained between 6.637‰ and 9.62‰. These diseases were the leading cause of death, accounting for over 99% of fatalities, emphasising the need for targeted prevention. Class C notifiable infectious diseases included 11 types, with 37 882 488 reported cases and an average incidence rate of 537.7343/100 000. The reported incidence rate increased moderately from 223.2087 to 298.0491/100 000 between 2020 and 2022. Following the relaxation of COVID-19 restrictions, it surged dramatically to a peak of 1128.668/100 000 in 2023 before declining to 790.0365/100 000 in 2024, indicating an overall elevated transmission level in the post-intervention period. There were 235 deaths, with a low average mortality rate of 0.0033/100 000, which showed minimal fluctuation throughout the five years. The CFR was negligible, averaging 0.006‰. These diseases accounted for over 70% of all reported cases but contributed to less than 0.2% of total deaths, highlighting effective management of milder cases (**Figure 1**, Panels F–H; Table S4 in the **Online Supplementary Document**).

### Analysis of notifiable infectious diseases based on transmission routes

Regarding transmission routes, RIDs accounted for the highest number of cases at 57.14% of the total, followed by IIDs at 23.02%, STBBIs at 18.35%, VBZDs at 0.82%, and diseases with other transmission routes at 0.66%. STBBIs were the leading cause of mortality, responsible for over 90.35% of deaths (**Figure 1**, Panels I–K; Table S5 in the **Online Supplementary Document**). Analysis of disease trends by transmission route showed distinct epidemiological characteristics (**Figure 1**, Panels L–N). There were 29 782 746 reported cases of RIDs. COVID-19 control measures were associated with a relatively low reported incidence rate (ranging from 104.1652 to 224.2876/100 000) from 2020 to 2022, but it spiked to 961.3799/100 000 in 2023 after restrictions eased, before declining to 683.6568/100 000 in 2024, indicating a sustained higher level of transmission compared to the pre-2023 period. The increase in reported incidence in 2023 likely stems from both a real resurgence in community transmission following the easing of NPIs and enhanced case detection due to greater healthcare utilisation, increased testing, and the resumption of routine surveillance. However, the current data cannot distinguish the relative impacts of these factors. The mortality rate varied from 0.1259 to 0.1592/100 000, and the CFR ranged from 0.166‰ to 1.208‰, both remaining relatively low. In total, 11 999 345 IIDs were reported, with reported incidence rates fluctuating between 121.1824 and 207.3271/100 000. The rates showed a bimodal pattern, peaking in 2021 and 2023. Mortality rate and CFR for IIDs remained low. There were 9 565 951 STBBI cases, with the reported incidence rates showing a slight increase from 123.1256/100 000 in 2020 to 146.86/100 000 in 2024. STBBIs were the leading cause of death among notifiable infectious diseases, with 106 877 deaths and a mortality rate averaging 1.5259/100 000. After the mortality rate peaked in 2023 at 1.7409/100 000, it slightly decreased in 2024 at 1.649/100 000. STBBIs had a higher CFR, ranging from 8.861‰ to 12.763‰, highlighting ongoing challenges in managing their rising incidence and high mortality. There were 429 420 reported VBZDs cases, with reported incidence rates ranging from 4.4752 to 7.3609/100 000. While the incidence rate slightly increased, the reported mortality rate remained low, ranging from 0.0108 to 0.0188/100 000. The CFR dropped from 4.20‰ in 2020 to 1.799‰ in 2024. Infectious diseases with other transmission routes were the least common, accounting for 343 200 cases, showing consistently low CFR, incidence, and mortality rates.

### Seasonal characteristics of notifiable infectious diseases

**Figure 2:**
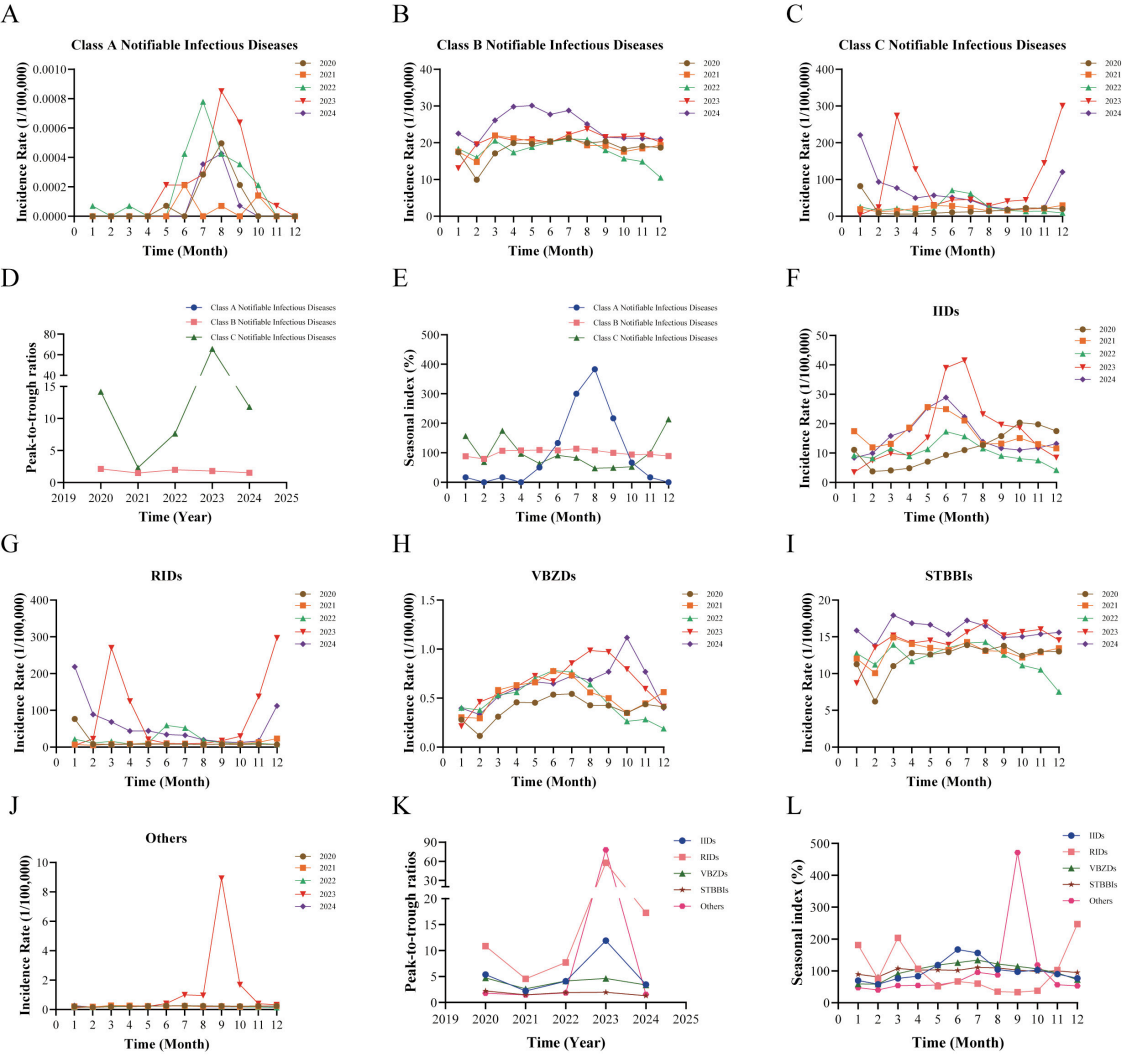
Monthly prevalence of notifiable infectious diseases in Mainland China (2020–2024). **Panel A.** Incidence rate of class A notifiable infectious diseases. **Panel B.** Incidence rate of class B notifiable infectious diseases. **Panel C.** Incidence rate of class C notifiable infectious diseases. **Panel D**. PTR for Class A, Class B, and Class C diseases. **Panel E**. SI for Class A, Class B, and Class C diseases. **Panel F.** Incidence rate of IID. **Panel G.** Incidence rate of RIDs. **Panel H.** Incidence rate of VBZDs. **Panel I.** Incidence rate of STBBIs. **Panel J.** Incidence rate of other notifiable infectious diseases. **Panel K**. PTR for IIDs, RIDs, VBZDs, STBBIs, and others. **Panel L**. SI for IIDs, RIDs, VBZDs, STBBIs, and others. IID – intestinal infectious disease, PTR – peak-to-trough ratio, RID – respiratory infectious disease, SI – seasonal index, STBBI – sexually transmitted and blood-borne infection, VBZD – vector-borne/zoonotic disease.

From 2020 to 2024, monthly reports of notifiable infectious diseases were consistently documented in mainland China (**Figure 2**, Panels A–E; Tables S6–8 in the **Online Supplementary Document**). Class A notifiable infectious diseases occurred sporadically, predominantly during the summer months, with the SI peaking in August (SI = 383.33) and July (SI = 300.00). We did not calculate the PTR due to the exceedingly low and sporadic incidence of cases. It is crucial to note that the exceptionally low and sporadic case counts lead to highly unstable rate estimates, necessitating cautious interpretation. Class B notifiable infectious diseases showed relatively stable incidence year-round, with the lowest rates consistently recorded in January and February. The PTR remained low throughout the study period, averaging 1.80, suggesting minimal seasonal variation at the aggregate level. The SI values ranged from 79.35 in February to 114.05 in July, further corroborating the lack of a pronounced seasonal pattern at the class level. This overall stability likely resulted from the amalgamation of diseases exhibiting opposing seasonal peaks, such as winter-peaking respiratory diseases and summer-peaking enteroviruses, along with those exhibiting minimal seasonality, such as chronic blood-borne diseases. Detailed seasonal patterns for specific diseases or transmission categories were elucidated in the subsequent route-based analysis. Class C notifiable infectious diseases showed moderate fluctuation from 2020 to 2022. However, following the easing of COVID-19 restrictions, a pronounced winter-spring peak became evident in 2023 and 2024, notably in December (SI = 213.66) and March (SI = 174.87). This transition was associated with a substantial increase in the PTR, which surged to 65.64 in 2023, compared to a five-year average of 20.32.

The analysis of transmission routes identified distinct patterns (**Figure 2**, Panels F–L; Tables S6–8 in the **Online Supplementary Document**). In general, IIDs typically peaked in summer (June–July), except in 2020, when the peak occurred in autumn (October–November). The average PTR was 5.40, indicating a moderate degree of seasonal fluctuation in incidence intensity. SI analysis further confirmed the summer peak pattern, with values peaking in June (SI = 167.21) and July (SI = 156.16). During the intervention period from 2020 to 2022, stringent NPIs implemented as part of COVID-19 measures disrupted the typical seasonal transmission patterns of RIDs, leading to the absence of a distinct seasonal pattern. Following the relaxation of COVID-19 measures in 2023–2024, RIDs exhibited a pronounced ‘suppression-rebound’ effect, characterised by a sharp and significant peak during the winter-spring season. The highest incidence rates during this period were observed in December (SI = 295.67) and March (SI = 244.52). This resurgence was further evidenced by a marked increase in the PTR, which reached 57.69 in 2023, compared with the five-year average of 19.62. Between 2020 and 2022, VBZD incidence consistently peaked from May to August each year, indicating a marked predominance during the warmer months. This pattern was supported by a moderate average PTR of 3.84 over the three-year period. SI analysis further substantiated this trend, identifying June (SI = 147.17) and July (SI = 143.51) as the months with the highest transmission intensity. However, a notable shift in this seasonal pattern was observed: the period of heightened incidence, which traditionally subsides after the summer, persisted into the autumn months in 2023. This recent extension of the transmission period has altered the overall seasonal dynamics, as reflected in both the PTR and SI analyses. STBBIs showed minimal seasonal variation, maintaining stable transmission year-round. Incidence rates fluctuated slightly each month, with a small decrease in cases during January and February. This was reflected in the lowest average PTR of 1.77 and SI values near 100, indicating no distinct seasonal peak. This stability aligns with the chronic nature of diseases such as HIV/acquired immune deficiency syndrome (AIDS) and syphilis, which are not influenced by seasonal factors. Infectious diseases with other transmission routes generally maintained a stable and low incidence rate, except for an anomalous increase observed from July to October 2023. This surge resulted in a significantly elevated PTR of 77.88 for that year and contributed to an increased five-year average PTR of 16.90.

### Analysis of the ranking of incidence and mortality rates

**Figure 3:**
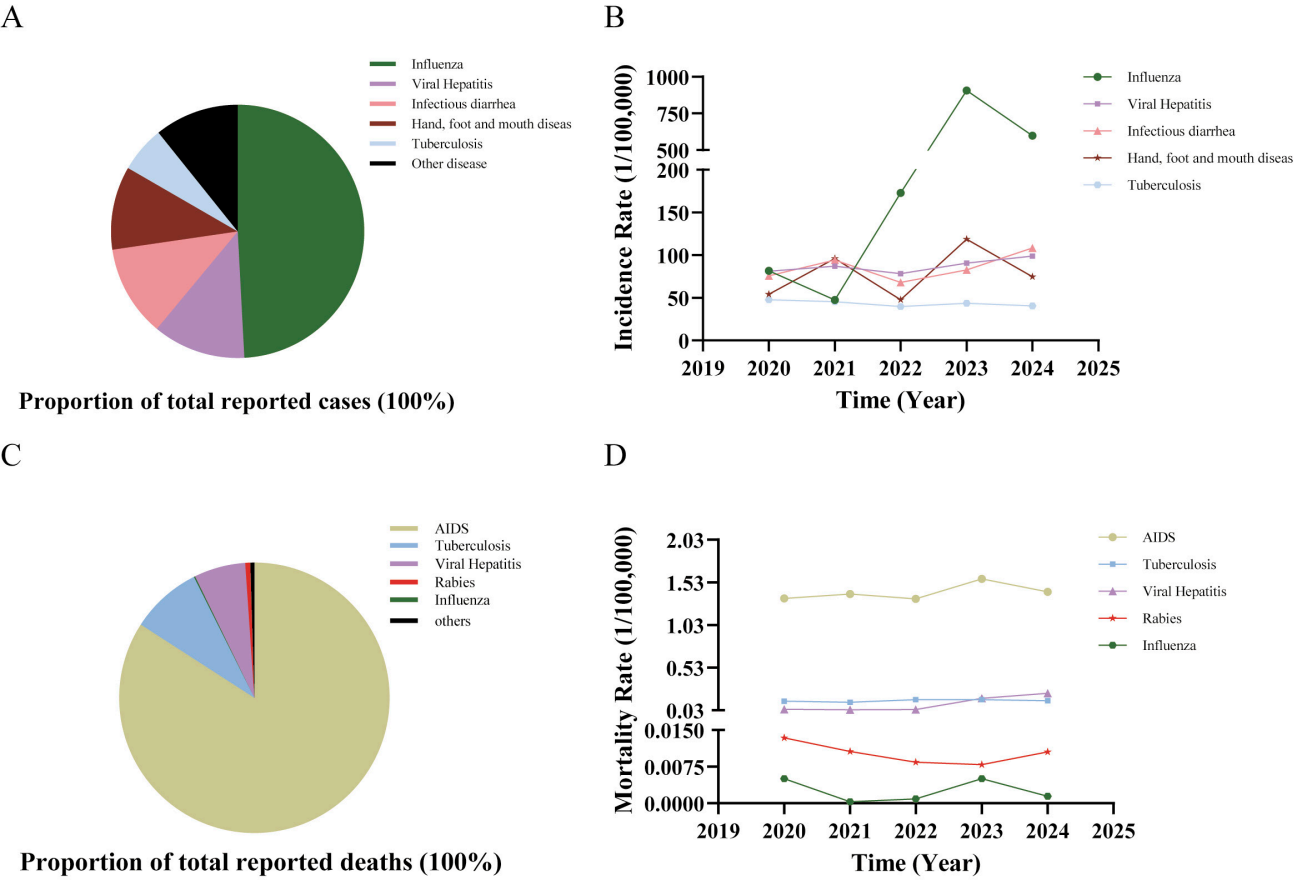
Top five notifiable infectious diseases by reported cases and mortality in Mainland China (2020–2024). **Panel A**. Proportion of total reported cases. **Panel B**. Incidence rates of key infectious diseases. **Panel C.** Proportion of total reported deaths. **Panel D.** Mortality rates of key infectious diseases.

Beyond temporal and seasonal patterns, the relative burden of different diseases, as reflected in incidence and mortality rankings, provides further insight into the evolving epidemiological landscape (Tables S9–14 in the **Online Supplementary Document**). Between 2020 and 2024, the top five notifiable infectious diseases in mainland China remained largely consistent, collectively accounting for 46 203 592 reported cases, representing 89.25% of all cases during this period. The highest number was of influenza cases (n = 25 454 008; 49.17%), followed by viral hepatitis (n = 6 140 406; 11.86%), infectious diarrhoea (n = 6 042 516; 11.67%), hand, foot, and mouth disease (n = 5 513 557; 10.65%), and tuberculosis (n = 3 053 105; 5.9%) (**Figure 3**, Panel A). Influenza had the highest annual incidence rate, except in 2021. Hand, foot, and mouth disease exhibited a unique bimodal fluctuation pattern, peaking in 2021 and 2023, ranking first and second, respectively. Viral hepatitis and infectious diarrhoea alternated between the second and third positions across most years, except in 2023, when diarrhoea dropped to fourth. Tuberculosis consistently ranked fifth, except in 2024, when it was overtaken by syphilis, and fell to sixth place (**Figure 3**, Panel B). A sensitivity analysis, excluding the highly variable influenza cases, was conducted to identify the leading contributors to morbidity independent of their influence. For the aggregate period 2020–2024, viral hepatitis emerged as the predominant cause of reported cases, followed by infectious diarrhoea, hand, foot and mouth disease, tuberculosis, and syphilis (Figure S1 in the **Online Supplementary Document**). The analyses showed that although influenza affected the rankings, the overall trends and conclusions regarding the prominence of other diseases remained consistent.

During the same period, a small number of diseases were responsible for most deaths (Tables S15–20 in the **Online Supplementary Document**). The leading causes were AIDS (84.12%), tuberculosis (8.5%), viral hepatitis (6.15%), rabies (0.6%), and influenza (0.15%). Together, these five diseases accounted for approximately 99.52% of all reported deaths (**Figure 3**, Panel C). Syphilis and epidemic haemorrhagic fever (HFRS) also contributed notably to mortality. AIDS consistently had the highest mortality rate each year. Tuberculosis and viral hepatitis alternated between the second and third place. Rabies was fourth, with influenza and HFRS alternating in fifth position annually, and syphilis entering the top five in 2024 (**Figure 3**, Panel D). The sensitivity analysis on mortality, excluding influenza, confirmed the stability of the leading causes of death. The top five ranking for the aggregate period remained AIDS, tuberculosis, viral hepatitis, rabies, and HFRS, with HFRS replacing influenza in the fifth position (Figure S1 in the **Online Supplementary Document**). This consistency highlights that the mortality structure was not primarily driven by influenza reporting dynamics.

### Analysis of the impact of excluding specific diseases

During the period from 2020 to 2024, a total of 1074 cases and three deaths from monkeypox and neonatal tetanus were reported, constituting a negligible portion of the total disease burden (Table S21 in the **Online Supplementary Document**). Notably, monkeypox was not classified as a legally notifiable infectious disease in mainland China until 20 September 2023, resulting in its absence from reports until that year, when 407 cases were documented, followed by 551 cases in 2024, with no associated fatalities. Neonatal tetanus cases remained consistently low, ranging from 19 to 34 annually, with a total of three deaths recorded from 2020 to 2022 and none from 2023 to 2024. In stark contrast, the remaining 38 notifiable infectious diseases reported during the same timeframe accounted for 51 771 730 cases and 118 206 deaths. The relatively small number of cases and deaths from these diseases, particularly in comparison to the overall burden, suggests that their exclusion does not detectably impact the observed trends, seasonal patterns, or mortality structure of the notifiable infectious diseases.

## DISCUSSION

The implementation of COVID-19 control and prevention measures in mainland China has profoundly and multifacetedly influenced the epidemiology of multiple notifiable infectious diseases, contributing to overall instability characterised by ‘suppression-rebound’ fluctuations [14–17]. We provide a comprehensive analysis of the characteristics and trends of 38 notifiable infectious diseases in mainland China from 2020 to 2024, excluding data on COVID-19, monkeypox, and neonatal tetanus to ensure methodological consistency and comparability. There were distinct epidemiological shifts driven by varying NPI intensities over the study period.

Between 2020 and 2024, 51 771 730 cases of notifiable infectious diseases were reported, with the incidence rate ranging from a low of 407.4242/100 000 in 2020 to a peak of 1326.8076/100 000 in 2023. The average incidence rate remained relatively low at 439.5573/100 000 from 2020 to 2022, representing an approximately 23.86% decline compared to the 2016–2019 pre-pandemic baseline of 577.2693/100 000 (Table S22 in the **Online Supplementary Document**). However, following the relaxation of NPIs in early 2023, the incidence rate rose sharply to 1326.8076/100 000, surpassing pre-pandemic levels before slightly declining to 1028.993/100 000 in 2024, indicating sustained elevated transmission. Furthermore, the composition of the disease spectrum and its temporal distribution have continued to be profoundly affected by the COVID-19 control and prevention measures [18]. This ‘suppression-rebound’ pattern underscores the transient impact of stringent NPIs and highlights the vulnerability of disease transmission to population mobility and social contact.

During the same period, the reported cases of Class A notifiable infectious diseases remained extremely rare, yet vigilance against importation risks remains necessary. Class B notifiable infectious diseases, although comprising only 26.83% of total cases, were the predominant contributors to mortality and responsible for over 99% of total deaths, with AIDS and viral hepatitis jointly accounting for more than 85% of these fatalities. The rise in both the incidence and mortality rates in 2023, and their persistence at elevated levels in 2024, indicated a continued need for enhanced prevention, especially for chronic infections with high CFR in the future. Conversely, Class C notifiable infectious diseases accounted for most reported cases (73.17%), consistent with findings from other studies [19,20], yet they contributed to less than 0.2% of deaths. Their incidence surged dramatically post-2022, with pronounced winter–spring peaks and a PTR reaching 65.64 in 2023, compared to an average of 8.06 during 2020–2022. This pattern indicates that while NPIs temporarily suppressed transmission, their relaxation led to a robust rebound, particularly for seasonal respiratory diseases.

Analysis by transmission routes further elucidated distinct epidemiological patterns. RIDs, accounting for 57.14% of cases, showed a ‘suppression-rebound’ trend associated with the implementation and subsequent easing of NPIs. Their spread was significantly reduced from 2020 to 2022, disrupting usual seasonal cycles. After policy changes in early 2023, there was a sharp resurgence with notable winter–spring peaks in December (SI = 295.67) and March (SI = 244.52) with a PTR of 57.69, much higher than the five-year average of 19.62. This indicates that diseases spread through droplets and contact are highly affected by population movement and intervention measures. In contrast, IIDs exhibited a consistent seasonal peak during the summer months, with the highest SI observed in June and July. This pattern reflects environmental and behavioural factors that persisted despite the overall impact of NPIs and was characterised by a moderate average PTR of 5.40. Conversely, STBBIs, which account for most mortality, showed minimal seasonal variation, with an average PTR of 1.77 and an SI near 100 throughout the year. This stability is consistent with their chronic nature and transmission modes, which are largely independent of climatic influences. VBZDs showed the anticipated predominance during the warmer seasons; however, activity extended into autumn 2023, potentially indicating changes in ecological or reporting dynamics. These route-specific seasonal patterns show that although NPIs broadly induced a ‘suppression-rebound’ effect, especially on respiratory and contact-driven diseases, the underlying seasonal transmission cycles persisted or reemerged after interventions. This highlights the importance of surveillance and control strategies that are responsive to sudden policy-induced changes while being rooted in the consistent seasonal behaviours of different pathogens.

Our ranking analysis further clarifies which diseases drove the observed trends in morbidity and mortality. From 2020 to 2024, influenza, viral hepatitis, infectious diarrhoea, hand, foot, and mouth disease, and tuberculosis collectively accounted for nearly 89.25% of all reported cases, while AIDS, tuberculosis, viral hepatitis, rabies, and influenza accounted for over 99% of deaths. This highlights the critical importance of targeting these diseases in prevention and control efforts. The sensitivity analysis, which excluded highly variable influenza cases, confirmed that viral hepatitis, tuberculosis, and AIDS remain the predominant contributors to the sustained disease burden, underscoring the need to prioritise these conditions in long-term control strategies. Between 2020 and 2022, the implementation of COVID-19 control measures led to a temporary disruption in the seasonal transmission patterns of respiratory viruses, a phenomenon consistent with other studies [21,22]. Subsequently, following the easing of restrictive containment measures, influenza activity during the 2023–2024 period rebounded to patterns similar to those observed in seasons preceding COVID-19 [23–25], although the current global landscape of influenza prevention and control remains complex and challenging. Concurrently, viral hepatitis and tuberculosis continue to pose significant public health challenges, maintaining high rankings in both incidence and mortality rates, which necessitate the ongoing implementation of comprehensive prevention and control strategies [26–31]. We identified AIDS as the leading cause of mortality during the study period. In mainland China, the reported mortality (ranging from 1.3369 to 1.5703/100 000) and incidence (ranging from 3.6853 to 4.4283/100 000) rates of AIDS from 2020 to 2024 were substantially lower than the mortality (3.0/100 000) [32] and incidence (5.1/100 000) [33] rates recorded in 2019, indicating the sustained efficacy of local prevention and control measures. However, these trends require cautious interpretation, as mortality reflects past infections and incidence can be influenced by testing and reporting practices. Although the observed declines align with intensified prevention efforts, the study design does not allow for definitive causal attribution to specific interventions. Globally, the situation remains severe, with approximately 39.9 million individuals living with HIV and about 630 000 AIDS-related deaths as of 2023 [34], indicating that substantial challenges persist in achieving the Joint United Nations Programme on HIV/AIDS target of eradicating AIDS by 2030 [35]. Furthermore, rabies continued to be an almost invariably fatal disease between 2020 and 2024, with a CFR of 91.56%, underscoring the critical importance of timely post-exposure prophylaxis for effective prevention [36].

We found that, influenced by COVID-19 control and prevention measures, the epidemiological profile of notifiable infectious diseases in mainland China from 2020 to 2024 displayed a distinctive pattern. This pattern is characterised by a persistent burden of chronic infectious diseases alongside a resurgence of acute RIDs. Chronic infectious diseases, including AIDS, viral hepatitis, and tuberculosis, continue to impose a significant health burden, necessitating long-term and stable resource allocation for their prevention and control. Meanwhile, seasonal RIDs, such as influenza, have exhibited abnormal epidemiological fluctuations in the post-pandemic era, emphasising the need for enhanced continuous monitoring of pathogen co-circulation and transmission dynamics. To achieve the strategic goal of ‘Healthy China 2030’, valuable lessons from the COVID-19 prevention and control should be integrated into routine public health measures. Additionally, tailored and comprehensive strategies should be employed based on the specific transmission pathways of different diseases, with particular emphasis on those with high incidence and mortality rates. This precision-based approach is crucial for sustainably mitigating the overall burden of infectious diseases. Such an approach will facilitate the transition of prevention and control strategies toward a model focused on ‘multi-disease co-prevention and systematic response’.

### Limitations

Despite the comprehensive nature of our analysis, several limitations should be acknowledged when interpreting the findings. First, we relied on publicly available surveillance data, which may be subject to reporting delays, underreporting, and diagnostic inaccuracies. We obtained the data from a passive reporting system that captures only diagnosed and reported cases, thereby missing mild or asymptomatic infections. Underreporting was likely more pronounced during the stringent COVID-19 control period (2020–2022), due to reduced healthcare access and resource diversion. After 2023, improved detection likely occurred alongside increased transmission. Therefore, the reported incidence trends reflected not only actual disease transmission but also variations in surveillance sensitivity, healthcare-seeking behaviour, and the intensity of testing and reporting. Second, to ensure data comparability, we excluded cases of COVID-19, monkeypox, and neonatal tetanus, potentially constraining a thorough evaluation of the overall burden of all notifiable infectious diseases. Third, we primarily focused on descriptive statistics and did not incorporate advanced modelling to adjust for confounding factors or quantify causal effects. Additionally, although we provided CIs for the monthly incidence rates, the estimates for certain strata, particularly Class A diseases, were based on very few cases, resulting in wide CIs that indicated substantial statistical uncertainty (Table S6 in the **Online Supplementary Document**). Similarly, CFRs for diseases with few deaths were highly unstable and should be interpreted with caution. Furthermore, although consistent with national surveillance conventions, categorising diseases by a single predominant transmission route may oversimplify the complex transmission dynamics of diseases that can spread through multiple pathways, potentially affecting the interpretation of trends within route-based categories.

Moreover, it is important to note that although we identified distinct periods of disease suppression (2020–2022) and rebound (2023–2024) in relation to shifts in COVID-19 policies, we did not explicitly categorise the entire study period into specific pandemic phases, such as the strict intervention phase, the policy transition phase, and the post-management adjustment phase. An analysis based on explicit phases could enhance the interpretability of epidemiological trends and clarify the ‘suppression-rebound’ dynamics associated with policy and behavioural changes. Also, the notable increase in RIDs observed in 2023 requires cautious interpretation, as it might be influenced by both enhanced detections resulting from increased healthcare utilisation and testing, as well as a genuine epidemiological resurgence attributable to the relaxation of COVID-19 restrictions. Our study design cannot disentangle these influences, and the absence of indicators such as outpatient visit volumes or test positivity rates limits a more granular analysis. Thus, while the temporal pattern suggests a ‘suppression-rebound’ effect, the observed trends likely arise from a combination of true transmission changes and surveillance artefacts. Future research should incorporate explicit phase definitions, vaccination coverage, population mobility metrics, and health care resource indicators to better understand the interplay between pandemic control measures and other infectious diseases. Meanwhile, developing comprehensive models that integrate climate, behavioural, and intervention-related factors would help quantify each factor's relative contribution to disease incidence.

## CONCLUSIONS

Between 2020 and 2022, the implementation of stringent COVID-19 control and prevention measures – such as social distancing, restrictions on gatherings, mask-wearing, and enhanced hand hygiene – resulted in a marked reduction in the incidence rates of various notifiable infectious diseases. This reduction was particularly evident for diseases transmitted via droplets and contact, primarily RIDs. These observations align with findings from multiple studies examining the impact of NPIs on infectious disease transmission [37–39]. However, following the reclassification of COVID-19 management from Class A to Class B in early 2023, the gradual return to regular social activities and increased population mobility caused a resurgence in the incidence rates of most infectious diseases [40], notably RIDs and some Class C diseases. This suggests that the COVID-19 containment measures exerted a ‘suppression-rebound’ effect on other infectious diseases. Therefore, in future responses to major emerging infectious diseases, it is crucial to comprehensively assess the collateral impact of control and prevention strategies on the overall disease burden.

## Additional material


Online Supplementary Document
