# Supplementary material for: Epidemic trends and characteristics of notifiable infectious diseases in mainland China during the COVID-19: a nationwide surveillance study, 2020–2024
Source: J Glob Health. 2026 Apr 10;16:04118. doi: 10.7189/jogh.16.04118 (PMC13065339; doi:10.7189/jogh.16.04118)
Supplement: Online Supplementary Document [file jogh-16-04118-s001.zip › jogh-16-04118-s001.pdf]

Supplement to: Xu J, Xu Y, Dai Z, Xu Z, Cai H, Zhao Z, Shang W, Dang Y. Epidemic trends and characteristics of notifiable infectious diseases in mainland China during the COVID-19: a nationwide surveillance study, 2020–2024. J Glob Health. 2026;16:04118.

Table S1. Classification, Main Transmission Routes, and Case Reporting of Notifiable Infectious Diseases in Mainland China, 2020–2024

Disease Name	Main transmission routes	case reports	Total number of cases (No)
**Class A notifiable infectious diseases**			
Plague	VBZDs	Yes	14
Cholera	IIDs	Yes	86
**Class B notifiable infectious diseases**			
SARS	RIDs	NO	0
AIDS	STBBIs	Yes	288213
Hepatitis		Yes	6140406
Hepatitis A	IIDs	Yes	63077
Hepatitis B	STBBIs	Yes	5296997
Hepatitis C	STBBIs	Yes	957793
Hepatitis D	STBBIs	Yes	1038
Hepatitis E	IIDs	Yes	134225
Other hepatitis	Others	Yes	36208
Poliomyelitis	IIDs	NO	0
Human infection with H5N1 virus	VBZDs	Yes	2
Measles	RIDs	Yes	3853
HFRS	VBZDs	Yes	32145
Rabies	VBZDs	Yes	781
Japanese encephalitis	VBZDs	Yes	971
Dengue	VBZDs	Yes	45185
Anthrax	VBZDs	Yes	1870
Dysentery	IIDs	Yes	215637
Tuberculosis	RIDs	Yes	3053105

Typhoid fever and paratyphoid fever	IIDs	Yes	30247
Meningococcal meningitis	RIDs	Yes	391
Pertussis	RIDs	Yes	570195
Diphtheria	RIDs	Yes	2
Neonatal tetanus	Others	Yes	Exclusion
Scarlet fever	RIDs	Yes	159113
Brucellosis	VBZDs	Yes	315644
Gonorrhea	STBBIs	Yes	534088
Syphilis	STBBIs	Yes	2487822
Leptospirosis	VBZDs	Yes	1618
Schistosomiasis	VBZDs	Yes	104
Malaria	VBZDs	Yes	7750
Human infection with H7N9 virus	VBZDs	NO	0
Monkeypox	STBBIs	Yes	Exclusion
COVID-19	RIDs	Yes	Exclusion
**Class C notifiable infectious diseases**			
Influenza	RIDs	Yes	25454008
Mumps	RIDs	Yes	536454
Rubella	RIDs	Yes	4808
Acute hemorrhagic conjunctivitis	Others	Yes	306992
Leprosy	RIDs	Yes	817
Typhus	VBZDs	Yes	6964
Kala azar	VBZDs	Yes	1187
Echinococcosis	VBZDs	Yes	15184
Filariasis	VBZDs	Yes	1
Infectious diarrhea	IIDs	Yes	6042516

Hand, foot and mouth disease	IIDs	Yes	5513557

Note: AIDS: Acquired immune deficiency syndrome; HFRS: Epidemic hemorrhagic fever; IIDs: Intestinal infectious diseases; Others: Infectious Diseases with Other Transmission Routes; RIDs: Respiratory Infectious Diseases; STBBIs: Sexually transmitted and blood-borne infections; VBZDs: Vector-Borne/Zoonotic Diseases. AIDS deaths refer to the total number of all-cause deaths reported monthly among AIDS patients. Infectious diarrhea cases exclude cholera, dysentery, typhoid, and paratyphoid fevers. The National Notifiable Disease Reporting System in China records both clinically diagnosed and laboratory-confirmed cases, including cause-specific deaths.

Table S2. Estimated Mid-Year Population in Mainland China, 2020–2024

**Year**	**Mid-year population (10,000)**
2020	141110
2021	141236
2022	141217.5
2023	141071
2024	140897.5

Table S3. Reported Incidence and Mortality of Notifiable Infectious Diseases in Mainland China, 2020–2024

**Year**	**Year-end total population (10,000)**	**Cases (No.)**	**Deaths (No.)**	**Incidence Rate (1/100,000)**	**Mortality Rate (1/100,000)**	**Case-Fatality Rate (‰)**
2019	141008	-	-	-	-	-
2020	141212	5719623	21739	407.4242	1.5485	3.801
2021	141260	6218271	22195	441.0813	1.5741	3.569
2022	141175	6641569	21860	470.1663	1.5475	3.291
2023	140967	18704666	26947	1326.8076	1.9116	1.441
2024	140828	14487601	25465	1028.9933	1.8091	1.758
Average annual	-	-	-	734.8945	1.6782	-
Total	705622	51771730	118206	-	-	2.283

Table S4. Epidemiological Characteristics of Notifiable Infectious Diseases Class A\B \C in Mainland China, 2020–2024

Year	2020	2021	2022	2023	2024	Average annual	Total
**Class A notifiable infectious diseases**							
Cases (No.)	15	6	33	34	12	-	100
Incidence Rate (1/100,000)	0.0011	0.0005	0.0023	0.0025	0.0008	0.0014	-
Deaths (No.)	3	0	1	1	1	-	6
Mortality Rate (1/100,000)	0.0002	0	0.0001	0.0001	0.0001	0.0001	-
Case-Fatality Rate (‰)	200	0	30.303	29.412	83.333	60	-
Proportion of Reported Cases (%)	0.0003	0.0001	0.0005	0.0002	0.0001	-	0.0002
Proportion of Reported Deaths (%)	0.0138	0	0.0046	0.0037	0.0039	-	0.0051
**Class B notifiable infectious diseases**							
Cases (No.)	2586108	2712016	2431294	2793236	3366488	-	13889142
Incidence Rate (1/100,000)	184.2144	192.3716	172.1148	198.1371	238.956	197.1588	-
Deaths (No.)	21651	22176	21832	26871	25435	-	117965
Mortality Rate (1/100,000)	1.5422	1.5728	1.5455	1.9062	1.807	1.6747	-
Case-Fatality Rate (‰)	8.372	8.177	8.98	9.62	6.637	8.183	-
Proportion of Reported Cases (%)	45.2147	43.6137	36.6072	14.9334	23.237	-	26.8277
Proportion of Reported Deaths (%)	99.5952	99.9144	99.8719	99.718	99.8822	-	99.7961
**Class C notifiable infectious diseases**							
Cases (No.)	3133500	3506249	4210242	15911396	11121101	-	37882488
Incidence Rate (1/100,000)	223.2087	248.7092	298.0491	1128.668	790.0365	537.7343	-
Deaths (No.)	85	19	27	75	29	-	235
Mortality Rate (1/100,000)	0.0061	0.0013	0.0019	0.0053	0.002	0.0033	-
Case-Fatality Rate (‰)	0.027	0.005	0.006	0.005	0.003	0.006	-
Proportion of Reported Cases (%)	54.7851	56.3862	63.3923	85.0665	76.7629	-	73.1722

Proportion of Reported Deaths (%)	0.391	0.0856	0.1235	0.2783	0.1139	-	0.1988

Table S5. Epidemiological Characteristics of Notifiable Infectious Diseases by Transmission Route in Mainland China, 2020–2024

**Year**	**2020**	**2021**	**2022**	**2023**	**2024**	**Average annual**	**Total**
**IIDs**							
Cases (No.)	1922323	2780018	1711822	2922792	2662390	-	11999345
Incidence Rate (1/100,000)	136.9328	197.1953	121.1824	207.3271	189.1345	170.54	-
Deaths (No.)	34	32	27	11	13	-	117
Mortality Rate (1/100,000)	0.0024	0.0023	0.0019	0.0008	0.0009	0.0018	-
Case-Fatality Rate (‰)	0.018	0.012	0.016	0.004	0.008	0.01	-
Proportion of Reported Cases (%)	33.61	44.71	24.49	15.63	18.38	-	23.02
Proportion of Reported Deaths (%)	0.16	0.14	0.12	0.04	0.05	-	0.1
**RIDs**							
Cases (No.)	1969284	1468498	3168287	13553053	9623624	-	29782746
Incidence Rate (1/100,000)	140.278	104.1652	224.2876	961.3799	683.6568	427.1696	-
Deaths (No.)	1995	1774	2226	2243	2046	-	10284
Mortality Rate (1/100,000)	0.1422	0.1259	0.1576	0.1592	0.1453	0.1670	-
Case-Fatality Rate (‰)	1.013	1.208	0.703	0.166	0.355	0.345	-
Proportion of Reported Cases (%)	34.43	23.62	45.32	72.46	66.43	-	57.14
Proportion of Reported Deaths (%)	9.18	7.99	10.14	8.32	8.03	-	8.69
**VBZDs**							
Cases (No.)	62851	85291	77432	103770	100076	-	429420
Incidence Rate (1/100,000)	4.4752	6.0499	5.4815	7.3609	6.9119	6.1954	-
Deaths (No.)	264	231	167	150	194	-	1006
Mortality Rate (1/100,000)	0.0188	0.0162	0.0118	0.0108	0.0139	0.0143	-
Case-Fatality Rate (‰)	4.2	2.708	2.157	1.446	1.799	2.343	-

Proportion of Reported Cases (%)	1.1	1.37	1.11	0.55	0.69	-	0.82
Proportion of Reported Deaths (%)	1.21	1.04	0.76	0.56	0.76	-	0.85
**STBBIs**							
Cases (No.)	1728491	1847230	1999907	1923020	2067303	-	9565951
Incidence Rate (1/100,000)	123.1256	131.0299	141.5763	136.4086	146.86	144.6202	-
Deaths (No.)	19443	20155	19524	24543	23212	-	106877
Mortality Rate (1/100,000)	1.385	1.4296	1.3821	1.7409	1.649	1.5259	-
Case-Fatality Rate (‰)	11.249	10.911	9.763	12.763	8.861	11.173	-
Proportion of Reported Cases (%)	30.22	29.71	28.61	10.28	14.27	-	18.35
Proportion of Reported Deaths (%)	89.44	90.81	88.96	91.08	91.15	-	90.35
**Others**							
Cases (No.)	36674	37234	33053	202031	34208	-	343200
Incidence Rate (1/100,000)	2.6124	2.6412	2.3399	14.331	2.4301	4.8907	-
Deaths (No.)	3	3	3	0	0	-	9
Mortality Rate (1/100,000)	0.0002	0.0002	0.0002	0	0	0.0001	-
Case-Fatality Rate (‰)	0.082	0.081	0.091	0	0.028	0.026	-
Proportion of Reported Cases (%)	0.64	0.6	0.47	1.08	0.24	-	0.66
Proportion of Reported Deaths (%)	0.01	0.01	0.01	0	0	-	0.01

Table S6. Monthly Incidence Rates of Notifiable Infectious Diseases by Legal Class and Transmission Route in Mainland China (2020–2024) with 95% Confidence Intervals

Year \Month	Jan.	Feb.	Mar.	Apr.	May.	Jun.	Jul.	Aug.	Sep.	Oct.	Nov.	Dec.
**Total of Notifiable Infectious Diseases (1/100,000)**												
2020	99.4142<br>(99.2499,99.5788)	18.3256<br>(18.255,18.3963)	22.895<br>(22.8161,22.9741)	25.9993<br>(25.9152,26.0835)	28.2157<br>(28.1282,28.3035)	31.1239<br>(31.032,31.2161)	33.3532<br>(33.258,33.4486)	33.6158<br>(33.5202,33.7115)	37.8909<br>(37.7894,37.9925)	40.6329<br>(40.5278,40.7382)	41.2968<br>(41.1908,41.4029)	38.3841<br>(38.282,38.4864)
2021	36.7219<br>(36.622,36.8219)	27.5615<br>(27.475,27.6482)	36.7055<br>(36.6057,36.8056)	42.5602<br>(42.4527,42.6679)	49.4925<br>(49.3766,49.6086)	48.3392<br>(48.2247,48.454)	44.4198<br>(44.31,44.5299)	33.9981<br>(33.902,34.0943)	35.3779<br>(35.2799,35.4762)	36.9539<br>(36.8537,37.0543)	39.9388<br>(39.8347,40.0432)	49.0934<br>(48.9779,49.209)

2022	44.1682<br>(44.0587,44.2779)	31.2846<br>(31.1924,31.377)	41.9693<br>(41.8625,42.0763)	29.4522<br>(29.3628,29.5419)	36.2284<br>(36.1292,36.3278)	90.946<br>(90.7889,91.1034)	82.9198<br>(82.7698,83.0701)	45.6278<br>(45.5165,45.7393)	33.9301<br>(33.8342,34.0263)	29.1659<br>(29.0769,29.2551)	28.6968<br>(28.6085,28.7853)	19.75<br>(19.6767,19.8234)
2023	17.6732<br>(17.6039,17.7427)	44.2814<br>(44.1717,44.3913)	295.6876<br>(295.4044,295.9711)	148.9432<br>(148.7421,149.1446)	51.5801<br>(51.4616,51.6987)	64.273<br>(64.1408,64.4054)	68.2488<br>(68.1125,68.3852)	52.1567<br>(52.0376,52.276)	62.3435<br>(62.2133,62.4739)	66.2817<br>(66.1474,66.4161)	166.7402<br>(166.5273,166.9532)	320.6235<br>(320.3286,320.9187)
2024	243.2661<br>(243.0089,243.5234)	113.1781<br>(113.0026,113.3538)	102.9186<br>(102.7513,103.0862)	79.5061<br>(79.359,79.6534)	87.1013<br>(86.9473,87.2554)	79.3027<br>(79.1558,79.4498)	72.6195<br>(72.4789,72.7603)	51.0751<br>(50.9572,51.1933)	41.8675<br>(41.7607,41.9744)	39.996<br>(39.8917,40.1006)	45.0947<br>(44.9839,45.2057)	141.5208<br>(141.3246,141.7173)
**By Legal Class**												
**Class A Notifiable Infectious Diseases (1/100,000)**												
2020	0	0	0	0	0.0001<br>(0,0.0004)	0	0.0003<br>(0.0001,0.0007)	0.0005<br>(0.0002,0.001)	0.0002<br>(0,0.0006)	0	0	0
2021	0	0	0	0	0	0.0002<br>(0,0.0006)	0	0.0001<br>(0,0.0004)	0	0.0001<br>(0,0.0005)	0	0
2022	0.0001<br>(0,0.0004)	0	0.0001<br>(0,0.0004)	0	0	0.0004<br>(0.0002,0.0009)	0.0008<br>(0.0004,0.0014)	0.0004<br>(0.0002,0.0009)	0.0004<br>(0.0001,0.0008)	0.0002(<br>0,0.0006)	0	0
2023	0	0	0	0	0.0002<br>(0,0.0006)	0.0002<br>(0,0.0006)	0.0003<br>(0.0001,0.0007)	0.0009<br>(0.0004,0.0015)	0.0006<br>(0.0003,0.0012)	0.0001<br>(0,0.0005)	0.0001<br>(0,0.0004)	0
2024	0	0	0	0	0	0	0.0004<br>(0.0001,0.0008)	0.0004<br>(0.0002,0.0009)	0.0001<br>(0,0.0004)	0	0	0
**Class B Notifiable Infectious Diseases (1/100,000)**												
2020	17.4034<br>(17.3347,17.4724)	9.957<br>(9.905,10.0092)	17.1096<br>(17.0414,17.1779)	19.9077<br>(19.8341,19.9814)	19.6917<br>(19.6185,19.765)	20.4<br>(20.3255,20.4746)	21.2577<br>(21.1817,21.3339)	19.9007<br>(19.8272,19.9745)	20.3786<br>(20.3042,20.4533)	18.284<br>(18.2135,18.3546)	19.0721<br>(19.0001,19.1443)	18.6787<br>(18.6075,18.7501)
2021	17.5667<br>(17.4976,17.636)	14.7823<br>(14.7189,14.8458)	21.9769<br>(21.8997,22.0543)	21.2243<br>(21.1484,21.3004)	20.4922<br>(20.4176,20.567)	20.3561<br>(20.2818,20.4307)	21.5106<br>(21.4342,21.5872)	19.3308<br>(19.2584,19.4035)	19.1925<br>(19.1203,19.2649)	17.5962<br>(17.527,17.6655)	18.4828<br>(18.412,18.5539)	19.3548<br>(19.2824,19.4275)
2022	18.3798<br>(18.3092,18.4506)	16.005<br>(15.9391,16.0711)	20.5751<br>(20.5003,20.65)	17.393<br>(17.3243,17.4619)	18.8542<br>(18.7826,18.9259)	20.2632<br>(20.189,20.3376)	21.0577<br>(20.982,21.1335)	20.8873<br>(20.812,20.9628)	17.9813<br>(17.9115,18.0514)	15.7016<br>(15.6363,15.7671)	14.8832<br>(14.8197,14.947)	10.5207<br>(10.4672,10.5743)
2023	13.0958<br>(13.0362,13.1557)	19.6181<br>(19.545,19.6913)	21.799<br>(21.722,21.8761)	20.6369<br>(20.562,20.712)	20.9638<br>(20.8883,21.0395)	20.107<br>(20.033,20.1811)	22.2563<br>(22.1785,22.3343)	23.7468<br>(23.6665,23.8274)	21.5586<br>(21.482,21.6353)	21.6716<br>(21.5948,21.7485)	21.9145<br>(21.8373,21.9919)	20.173<br>(20.099,20.2473)

2024	22.5256<br>(22.4473,22.6041)	19.561<br>(19.488,19.6341)	26.132<br>(26.0476,26.2165)	29.8215<br>(29.7314,29.9118)	30.1071<br>(30.0165,30.1978)	27.7243<br>(27.6375,27.8114)	28.7978<br>(28.7093,28.8865)	25.0981<br>(25.0155,25.181)	21.5602<br>(21.4836,21.637)	21.2989<br>(21.2228,21.3752)	21.1594<br>(21.0835,21.2355)	20.9771<br>(20.9015,21.0528)
**Class C Notifiable Infectious Diseases (1/100,000)**												
2020	82.0108<br>(81.8615,82.1603)	8.3686<br>(8.3209,8.4164)	5.7854<br>(5.7458,5.8252)	6.0916<br>(6.051,6.1325)	8.524<br>(8.4759,8.5723)	10.724<br>(10.67,10.7781)	12.0952<br>(12.0379,12.1527)	13.7145<br>(13.6535,13.7758)	17.512<br>(17.443,17.5812)	22.3489<br>(22.271,22.4271)	22.2247<br>(22.147,22.3026)	19.7054<br>(19.6322,19.7788)
2021	19.1552<br>(19.0831,19.2275)	12.7792<br>(12.7203,12.8383)	14.7286<br>(14.6654,14.792)	21.3358<br>(21.2597,21.4122)	29.0003<br>(28.9115,29.0892)	27.9829<br>(27.8957,28.0702)	22.9092<br>(22.8304,22.9883)	14.6672<br>(14.6041,14.7304)	16.1855<br>(16.1192,16.2519)	19.3576<br>(19.2851,19.4303)	21.456<br>(21.3797,21.5325)	29.7385<br>(29.6487,29.8286)
2022	25.7883<br>(25.7046,25.8722)	15.2796<br>(15.2152,15.3442)	21.3942<br>(21.3179,21.4706)	12.0592<br>(12.002,12.1166)	17.3742<br>(17.3055,17.4431)	70.6824<br>(70.5438,70.8211)	61.8614<br>(61.7318,61.9912)	24.7401<br>(24.6581,24.8222)	15.9484<br>(15.8827,16.0144)	13.4641<br>(13.4036,13.5247)	13.8136<br>(13.7524,13.875)	9.2293<br>(9.1793,9.2796)
2023	4.5774<br>(4.5422,4.6129)	24.6633<br>(24.5815,24.7454)	273.8887<br>(273.616,274.1615)	128.3063<br>(128.1196,128.4933)	30.6161<br>(30.5248,30.7075)	44.1658<br>(44.0563,44.2756)	45.9922<br>(45.8803,46.1042)	28.409<br>(28.3212,28.4971)	40.7843<br>(40.679,40.8898)	44.6099<br>(44.4998,44.7203)	144.8256<br>(144.6272,145.0242)	300.4505<br>(300.1649,300.7362)
2024	220.7405<br>(220.4955,220.9857)	93.6171<br>(93.4575,93.777)	76.7867<br>(76.6421,76.9314)	49.6846<br>(49.5683,49.8011)	56.9942<br>(56.8696,57.119)	51.5783<br>(51.4599,51.697)	43.8213<br>(43.7121,43.9307)	25.9766<br>(25.8925,26.0609)	20.3072<br>(20.2328,20.3817)	18.6971<br>(18.6258,18.7687)	23.9353<br>(23.8546,24.0162)	120.5437<br>(120.3626,120.725)
**By Transmission Route**												
**IIDs (1/100,000)**												
2020	11.0926<br>(11.0377,11.1476)	3.7835<br>(3.7515,3.8157)	4.0948<br>(4.0615,4.1283)	4.8596<br>(4.8233,4.8961)	7.0886<br>(7.0447,7.1326)	9.348<br>(9.2976,9.3985)	11.0102<br>(10.9555,11.0651)	12.7429<br>(12.6841,12.8019)	15.7641<br>(15.6986,15.8297)	20.3558<br>(20.2814,20.4303)	19.7855<br>(19.7122,19.859)	17.4793<br>(17.4104,17.5485)
2021	17.4259<br>(17.3571,17.4948)	11.9318<br>(11.8749,11.9889)	13.1619<br>(13.1021,13.2218)	18.6443<br>(18.5731,18.7156)	25.6434<br>(25.56,25.727)	24.9254<br>(24.8431,25.0078)	21.0674<br>(20.9918,21.1433)	13.0489<br>(12.9894,13.1086)	13.2105<br>(13.1506,13.2706)	15.1237(15.0596,1<br>5.188)	13.0345<br>(12.975,13.0942)	11.588<br>(11.5319,11.6443)
2022	9.2487<br>(9.1986,9.299)	8.233<br>(8.1858,8.2805)	11.5615<br>(11.5054,11.6177)	8.9372<br>(8.888,8.9866)	11.3272<br>(11.2718,11.3829)	17.3163<br>(17.2477,17.385)	15.6879<br>(15.6227,15.7534)	11.56<br>(11.504,11.6162)	9.0656<br>(9.016,9.1154)	8.1172<br>(8.0703,8.1643)	7.5401<br>(7.4949,7.5856)	4.2435<br>(4.2095,4.2776)
2023	3.4882<br>(3.4575,3.5192)	7.5119<br>(7.4667,7.5573)	9.9003<br>(9.8485,9.9524)	9.2529<br>(9.2027,9.3032)	15.2659<br>(15.2015,15.3305)	38.9862<br>(38.8832,39.0893)	41.5114<br>(41.4051,41.6178)	23.2435<br>(23.164,23.3232)	19.6631<br>(19.59,19.7365)	18.6678<br>(18.5966,18.7393)	12.2589<br>(12.2012,12.3168)	8.4595<br>(8.4116,8.5076)
2024	8.3974<br>(8.3496,8.4454)	9.9342<br>(9.8823,9.9864)	15.8023<br>(15.7368,15.8681)	17.9979<br>(17.9279,18.0681)	25.3611<br>(25.278,25.4443)	28.8966<br>(28.8079,28.9855)	22.3025<br>(22.2246,22.3806)	13.8738<br>(13.8123,13.9354)	11.6352<br>(11.5789,11.6917)	11.0293<br>(10.9745,11.0843)	11.752<br>(11.6955,11.8088)	13.174<br>(13.1142,13.2341)
**RIDs (1/100,000)**												
2020	76.559	8.066	7.2836	7.682	7.844	8.0556	7.6725	7.0461	7.6807	7.2799	7.8155	7.2499

	(76.4148,76.7034)	(8.0192,8.113)	(7.2391,7.3283)	(7.6364,7.7279)	(7.7979,7.8904)	(8.0088,8.1025)	(7.6268,7.7183)	(7.0024,7.0901)	(7.6351,7.7266)	(7.2354,7.3245)	(7.7694,7.8617)	(7.2055,7.2944)
2021	6.7304	5.0936	7.7732	8.9846	9.4596	9.175	8.0786	7.0976	8.3867	9.1003	13.3426	23.2418
	(6.6876,6.7733)	(5.0564,5.131)	(7.7272,7.8193)	(8.9352,9.0342)	(9.409,9.5105)	(9.1251,9.2251)	(8.0318,8.1256)	(7.0537,7.1416)	(8.339,8.4346)	(9.0506,9.1502)	(13.2824,13.4029)	(23.1624,23.3214)
2022	21.5782	11.2936	15.7278	8.0701	11.3516	59.08	52.0834	18.9357	11.6727	9.4859	10.197	7.6598
	(21.5017,21.6549)	(11.2382,11.3491)	(15.6625,15.7933)	(8.0233,8.1171)	(11.2961,11.4073)	(58.9533,59.2069)	(51.9645,52.2026)	(18.864,19.0076)	(11.6164,11.7292)	(9.4351,9.5368)	(10.1444,10.2498)	(7.6142,7.7056)
2023	5.1468	22.5594	269.8576	124.7083	20.864	10.2938	9.2405	10.0128	17.5748	29.4664	137.4721	296.899
	(5.1095,5.1844)	(22.4811,22.6379)	(269.5869,270.1284)	(124.5242,124.8926)	(20.7887,20.9395)	(10.2409,10.3469)	(9.1904,9.2908)	(9.9606,10.0651)	(17.5057,17.6441)	(29.3769,29.5561)	(137.2788,137.6655)	(296.6152,297.1831)
2024	218.3511	88.9515	68.4344	43.8169	44.1775	34.1675	32.1258	19.8543	14.3605	12.6539	17.0295	112.1627
	(218.1075,218.595)	(88.7959,89.1073)	(68.2979,68.571)	(43.7077,43.9263)	(44.0678,44.2874)	(34.071, 34.2641)	(32.0323,32.2195)	(19.7808,19.928)	(14.298,14.4232)	(12.5952,12.7128)	(16.9614,17.0977)	(111.988,112.3376)
**VBZDs (1/100,000)**												
2020	0.2799	0.1146	0.311	0.4574	0.4531	0.5357	0.5428(0.5307,0.5	0.4273	0.4238	0.3499	0.4364	0.4101
	(0.2713,0.2888)	(0.1091,0.1203)	(0.3019,0.3204)	(0.4463,0.4687)	(0.442,0.4643)	(0.5237,0.5479)	551)	(0.4166,0.4383)	(0.4131,0.4347)	(0.3402,0.3598)	(0.4256,0.4474)	(0.3996,0.4208)
2021	0.305	0.2941	0.5816	0.6314	0.6608	0.776	0.732	0.5586	0.4995	0.35	0.4459	0.5602
	(0.2959,0.3142)	(0.2852,0.3032)	(0.5691,0.5943)	(0.6183,0.6446)	(0.6475,0.6744)	(0.7615,0.7907)	(0.718,0.7463)	(0.5464,0.5711)	(0.4879,0.5113)	(0.3403,0.3599)	(0.435,0.4571)	(0.5479,0.5727)
2022	0.4021	0.3786	0.5291	0.561	0.7013	0.7813	0.7661	0.6399	0.4344	0.2631	0.2828	0.1891
	(0.3918,0.4127)	(0.3686,0.3889)	(0.5172,0.5412)	(0.5487,0.5735)	(0.6876,0.7153)	(0.7668,0.7961)	(0.7517,0.7806)	(0.6267,0.6532)	(0.4236,0.4454)	(0.2547,0.2717)	(0.2741,0.2917)	(0.182,0.1965)
2023	0.2133	0.4608	0.5349	0.6183	0.7274	0.6719	0.8552	0.9852	0.9709	0.7948	0.5949	0.4115
	(0.2057,0.2211)	(0.4496,0.4721)	(0.5229,0.5471)	(0.6054,0.6315)	(0.7134,0.7416)	(0.6585,0.6856)	(0.84,0.8706)	(0.9689,1.0017)	(0.9547,0.9873)	(0.7801,0.8096)	(0.5823,0.6078)	(0.401,0.4222)
2024	0.3965	0.3315	0.5155	0.5961	0.665	0.6466	0.7262	0.6844	0.7683	1.1167	0.7677	0.3989
	(0.3862,0.4071)	(0.3221,0.3412)	(0.5037,0.5275)	(0.5834,0.609)	(0.6516,0.6786)	(0.6334,0.6601)	(0.7122,0.7404)	(0.6708,0.6982)	(0.7539,0.7829)	(1.0993,1.1343)	(0.7533,0.7823)	(0.3885,0.4094)
**STBBIs (1/100,000)**												
2020	11.2689	6.2144	11.02	12.7856	12.6018	12.9294	13.8677	13.1508	13.7818	12.4199	13.0234	13.0081
	(11.2136,11.3245)	(6.1733,6.2556)	(10.9653,11.0749)	(12.7267,12.8448)	(12.5433,12.6605)	(12.8702,12.9889)	(13.8063,13.9293)	(13.091,13.2108)	(13.7206,13.8432)	(12.3618,12.4782)	(12.9639,13.0831)	(12.9487,13.0678)
2021	12.0478	10.0588	14.9209	14.0403	13.4872	13.2363	14.295	13.0919	13.0581	12.1853	12.9046	13.4643
	(11.9906,12.1052)	(10.0066,10.1113)	(14.8573,14.9848)	(13.9785,14.1022)	(13.4267,13.5479)	(13.1764,13.2964)	(14.2327,14.3575)	(13.0323,13.1517)	(12.9985,13.1178)	(12.1278,12.243)	(12.8455,12.964)	(13.4038,13.5249)
2022	12.7626	11.2287	13.9241	11.6726	12.6251	13.5191	14.1521	14.2852	12.5547	11.1192	10.517	7.5219
	(12.7037,12.8216)	(11.1735,11.2841)	(13.8626,13.9857)	(11.6164,11.7291)	(12.5666,12.6839)	(13.4586,13.5799)	(14.0901,14.2142)	(14.2229,14.3477)	(12.4963,12.6133)	(11.0643,11.1744)	(10.4636,10.5707)	(7.4767,7.5672)

2023	8.7103 (8.6617,8.7591)	13.5635 (13.5028,13.6244)	15.1871 (15.1229,15.2515)	14.1638 (14.1018,14.226)	14.5109 (14.4481,14.5739)	13.9206 (13.8591,13.9823)	15.6418 (15.5766,15.7072)	16.9577 (16.8898,17.0258)	15.2099 (15.1456,15.2743)	15.6688 (15.6035,15.7342)	16.0215 (15.9555,16.0877)	14.5356 (14.4727,14.5986)
2024	15.8606 (15.7949,15.9265)	13.7942 (13.733,13.8557)	17.9285 (17.8587,17.9985)	16.8557 (16.7879,16.9236)	16.6476 (16.5803,16.7151)	15.3441 (15.2795,15.4089)	17.2198 (17.1513,17.2884)	16.471 (16.404,16.5381)	14.917 (14.8533,14.9809)	15.0283 (14.9644,15.0924)	15.3755 (15.3108,15.4404)	15.6125 (15.5473,15.6779)
**Others (1/100,000)**												
2020	0.2138 (0.2062,0.2216)	0.1471 (0.1409,0.1536)	0.1855 (0.1785,0.1928)	0.2147 (0.2071,0.2224)	0.2283 (0.2204,0.2363)	0.2553 (0.2471,0.2638)	0.26 (0.2517,0.2686)	0.2486 (0.2404,0.257)	0.2405 (0.2324,0.2487)	0.2276 (0.2197,0.2356)	0.2361 (0.2281,0.2442)	0.2366 (0.2287,0.2448)
2021	0.2129 (0.2054,0.2207)	0.1831 (0.1761,0.1903)	0.268 (0.2595,0.2767)	0.2597 (0.2514,0.2683)	0.2414 (0.2334,0.2497)	0.2266 (0.2188,0.2346)	0.2468 (0.2386,0.2551)	0.2011 (0.1938,0.2086)	0.2232 (0.2154,0.2311)	0.1946 (0.1874,0.2021)	0.2112 (0.2037,0.2189)	0.2391 (0.2311,0.2473)
2022	0.1765 (0.1697,0.1836)	0.1506 (0.1443,0.1572)	0.2269 (0.2191,0.2349)	0.2113 (0.2038,0.219)	0.2231 (0.2154,0.2311)	0.2493 (0.2411,0.2576)	0.2304 (0.2225,0.2384)	0.2071 (0.1996,0.2147)	0.2027 (0.1954,0.2103)	0.1805 (0.1736,0.1876)	0.1598(0.1533,0.1666)	0.1357 (0.1297,0.1419)
2023	0.1146 (0.109,0.1203)	0.1858 (0.1787,0.193)	0.2077 (0.2002,0.2154)	0.1999 (0.1926,0.2074)	0.2118 (0.2043,0.2195)	0.4005 (0.3901,0.4111)	0.9999 (0.9834,1.0165)	0.9576 (0.9415,0.9739)	8.9247 (8.8755,8.9742)	1.6839 (1.6626,1.7055)	0.3928 (0.3825,0.4033)	0.3179 (0.3087,0.3274)
2024	0.2604 (0.252,0.269)	0.1666 (0.1599,0.1735)	0.2316 (0.2237,0.2397)	0.2395 (0.2315,0.2478)	0.2501 (0.2419,0.2585)	0.2479 (0.2398,0.2563)	0.2451 (0.237,0.2535)	0.1917 (0.1845,0.1991)	0.1864 (0.1794,0.1937)	0.1679 (0.1612,0.1748)	0.1701 (0.1633,0.177)	0.1727 (0.166,0.1798)

Table S7. Peak-to-Trough Ratio of Notifiable Infectious Diseases by Legal Class and Transmission Route in Mainland China, 2020–2024

Disease Classification	2020	2021	2022	2023	2024	Average
**By Legal Class**						
Class A Notifiable Infectious Diseases	-	-	-	-	-	-
Class B Notifiable Infectious Diseases	2.13	1.49	2	1.81	1.54	1.8
Class C Notifiable Infectious Diseases	14.18	2.33	7.66	65.64	11.81	20.32
**By Transmission Route**						
IIDs	5.38	2.21	4.08	11.9	3.44	5.4
RIDs	10.87	4.56	7.71	57.69	17.26	19.62
VBZDs	4.74	2.64	4.13	4.62	3.37	3.9

STBBIs	2.23	1.48	1.9	1.95	1.3	1.77
Others	1.77	1.46	1.84	77.88	1.56	16.9

Table S8. Seasonal Index of Notifiable Infectious Diseases by Legal Class and Transmission Route in Mainland China, 2020–2024

Disease Classification	Jan.	Feb.	Mar.	Apr.	May.	Jun.	Jul.	Aug.	Sep.	Oct.	Nov.	Dec.
**By Legal Class**												
Class A Notifiable Infectious Diseases	16.67	0	16.67	0	50	133.33	300.00	383.33	216.67	66.67	16.67	0
Class B Notifiable Infectious Diseases	88.33	79.35	106.82	108.2	109.32	108.07	114.05	108.18	99.95	93.87	94.82	89.06
Class C Notifiable Infectious Diseases	156.91	68.91	174.87	96.87	63.48	91.37	83.15	47.89	49.33	52.77	100.78	213.66
**By Transmission Route**												
IIDs	69.49	57.93	76.3	83.54	118.52	167.21	156.16	104.22	97.04	102.58	90.09	76.9
RIDs	181.25	75.05	203.72	106.68	51.72	66.66	60.28	34.74	32.94	37.53	102.59	246.85
RIDs (2023-2024)	161.55	80.6	244.52	121.81	47.01	32.14	29.9	21.59	23.08	30.45	111.68	295.67
VBZDs	58.93	58.29	91.23	105.7	118.37	125.89	133.67	121.61	114.28	106.08	93.28	72.69
VBZDs (2020-2022)	69.4	55.36	99.97	116.01	127.64	147.17	143.51	114.32	95.47	67.71	81.92	81.52
STBBIs	89.42	80.88	107.6	102.5	103.02	101.66	110.84	109.04	102.5	97.93	100.03	94.57
Others	47.17	40.18	54	54.26	55.69	66.53	95.59	87.1	471.53	118.37	56.42	53.15

Table S9. Top Five Notifiable Infectious Diseases by Cumulative Reported Incidence Rate in Mainland China, 2020-2024

Rank	2020	2021	2022	2023	2024	(2020-2024)
1	Influenza	Hand, foot and mouth disease	Influenza	Influenza	Influenza	Influenza
2	Viral Hepatitis	Infectious diarrhea	Viral Hepatitis	Hand, foot and mouth disease	Infectious diarrhea	Viral Hepatitis

3	Infectious diarrhea	Viral Hepatitis	Infectious diarrhea	Viral Hepatitis	Viral Hepatitis	Infectious diarrhea
4	Hand, foot and mouth disease	Influenza	Hand, foot and mouth disease	Infectious diarrhea	Hand, foot and mouth disease	Hand, foot and mouth disease
5	Tuberculosis	Tuberculosis	Tuberculosis	Tuberculosis	Syphilis	Tuberculosis

Table S10. Ranking of the Top 5 Notifiable Infectious Diseases by Total Cases in Mainland China, 2020–2024

Disease Name	Cases (No.)	Deaths (No.)	Five-year total reported Incidence Rate (1/100,000)	Five-year total reported Mortality Rate (1/100,000)	Case Proportion (%)
Influenza	25454008	177	1806.4428	0.0126	49.17
Viral Hepatitis	6140406	7273	435.8926	0.5163	11.86
Infectious diarrhea	6042516	23	428.961	0.0015	11.67
Hand, foot and mouth disease	5513557	18	391.3409	0.0013	10.65
Tuberculosis	3053105	10043	216.7492	0.7129	5.9
Other disease	5568138	100672	395.0862	7.1462	10.76

Table S11. Ranking of the Top 5 Notifiable Infectious Diseases by Reported Incidence Rates in Mainland China, 2020–2024

Year	Influenza (1/100,000)	Viral Hepatitis (1/100,000)	Infectious diarrhea (1/100,000)	Hand, foot and mouth disease (1/100,000)	Tuberculosis (1/100,000)
2020	81.5816	81.1188	75.6692	54.2336	47.7644
2021	47.4008	86.9757	94.3262	96.0823	45.3651
2022	172.9291	78.2858	67.934	47.6363	39.7032
2023	906.5644	90.6879	82.6598	118.7077	43.4893

2024	597.9669	98.8244	108.3718	74.681	40.4272

Table 12. Top Five Notifiable Infectious Diseases (Excluding Influenza) by Cumulative Reported Incidence Rate in Mainland China, 2020-2024

Rank	2020	2021	2022	2023	2024	Aggregate (2020-2024)
1	Viral Hepatitis	Hand, foot and mouth disease	Viral Hepatitis	Hand, foot and mouth disease	Infectious diarrhea	Viral Hepatitis
2	Infectious diarrhea	Infectious diarrhea	Infectious diarrhea	Viral Hepatitis	Viral Hepatitis	Infectious diarrhea
3	Hand, foot and mouth disease	Viral Hepatitis	Hand, foot and mouth disease	Infectious diarrhea	Hand, foot and mouth disease	Hand, foot and mouth disease
4	Tuberculosis	Tuberculosis	Tuberculosis	Tuberculosis	Syphilis	Tuberculosis
5	Syphilis	Syphilis	Syphilis	Syphilis	Tuberculosis	Syphilis

Table S13. Ranking of the Top 5 Notifiable Infectious Diseases (Excluding Influenza) by Total Cases in Mainland China, 2020–2024

Disease Name	Cases (No.)	Deaths (No.)	Five-year total reported Incidence Rate (1/100,000)	Five-year total reported Mortality Rate (1/100,000)	Case Proportion (%)
Viral Hepatitis	6140406	7273	435.8926	0.5163	11.86
Infectious diarrhea	6042516	23	428.961	0.0015	11.67
Hand, foot and mouth disease	5513557	18	391.3409	0.0013	10.65
Tuberculosis	3053105	10043	216.7492	0.7129	5.9
Syphilis	2487822	150	176.6074	0.0105	4.81
Other disease	28534324	100699	2024.9216	7.1483	55.12

Table S14. Ranking of the Top 5 Notifiable Infectious Diseases (Excluding Influenza) by Reported Incidence Rates in Mainland China, 2020–2024

Year	Viral Hepatitis (1/100,000)	Infectious diarrhea (1/100,000)	Hand, foot and mouth disease (1/100,000)	Tuberculosis (1/100,000)	Syphilis (1/100,000)
2020	81.1188	75.6692	54.2336	47.7644	33.0831
2021	86.9757	94.3262	96.0823	45.3651	34.0493
2022	78.2858	67.934	47.6363	39.7032	31.2303
2023	90.6879	82.6598	118.7077	43.4893	37.6036
2024	98.8244	108.3718	74.681	40.4272	40.6411

Table 15. Top Five Notifiable Infectious Diseases by Cumulative Reported Mortality Rate in Mainland China, 2020-2024

Rank	2020	2021	2022	2023	2024	Aggregate (2020-2024)
1	AIDS	AIDS	AIDS	AIDS	AIDS	AIDS
2	Tuberculosis	Tuberculosis	Tuberculosis	Viral Hepatitis	Viral Hepatitis	Tuberculosis
3	Viral Hepatitis	Viral Hepatitis	Viral Hepatitis	Tuberculosis	Tuberculosis	Viral Hepatitis
4	Rabies	Rabies	Rabies	Rabies	Rabies	Rabies
5	Influenza	HFRS	HFRS	Influenza	Pertussis	Influenza

Table S16. Ranking of the Top 5 Notifiable Infectious Diseases by Total deaths in Mainland China, 2020–2024

Disease Name	Cases (No.)	Deaths (No.)	Five-year total reported Incidence Rate (1/100,000)	Five-year total reported Mortality Rate (1/100,000)	Case Proportion (%)

AIDS	288213	99434	20.4611	7.0583	84.12
Tuberculosis	3053105	10043	216.7492	0.7129	8.5
Viral Hepatitis	6140406	7273	435.8926	0.5163	6.15
Rabies	781	715	0.0555	0.0508	0.6
Influenza	25454008	177	1806.4428	0.0126	0.15
Other disease	16835217	564	1194.8715	0.0399	0.48

Table S17. Ranking of the Top 5 Notifiable Infectious Diseases by Reported Mortality Rates in Mainland China, 2020–2024

**Year**	**AIDS (1/100,000)**	**Tuberculosis (1/100,000)**	**Viral Hepatitis (1/100,000)**	**Rabies (1/100,000)**	**Influenza (1/100,000)**
2020	1.3405	0.1367	0.0419	0.0134	0.005
2021	1.3919	0.1251	0.0369	0.0106	0.0003
2022	1.3369	0.1561	0.0384	0.0084	0.0009
2023	1.5703	0.1537	0.17	0.0079	0.005
2024	1.4187	0.1413	0.2291	0.0105	0.0014

Table 18. Top Five Notifiable Infectious Diseases (Excluding Influenza) by Cumulative Reported Mortality Rate in Mainland China, 2020-2024

Rank	**2020**	**2021**	**2022**	**2023**	**2024**	**Aggregate (2020-2024)**
1	AIDS	AIDS	AIDS	AIDS	AIDS	AIDS
2	Tuberculosis	Tuberculosis	Tuberculosis	Viral Hepatitis	Viral Hepatitis	Tuberculosis
3	Viral Hepatitis	Viral Hepatitis	Viral Hepatitis	Tuberculosis	Tuberculosis	Viral Hepatitis
4	Rabies	Rabies	Rabies	Rabies	Rabies	Rabies

5	Syphilis	HFRS	HFRS	Syphilis	Pertussis	HFRS

Table S19. Ranking of the Top 5 Notifiable Infectious Diseases (Excluding Influenza) by Total deaths in Mainland China, 2020–2024

Disease Name	Cases (No.)	Deaths (No.)	Five-year total reported Incidence Rate (1/100,000)	Five-year total reported Mortality Rate (1/100,000)	Case Proportion (%)
AIDS	288213	99434	20.4611	7.0583	84.12
Tuberculosis	3053105	10043	216.7492	0.7129	8.5
Viral Hepatitis	6140406	7273	435.8926	0.5163	6.15
Rabies	781	715	0.0555	0.0508	0.6
HFRS	32145	170	2.2824	0.0121	0.14
Other disease	42257080	571	2999.0319	0.0404	0.49

Table S20. Ranking of the Top 5 Notifiable Infectious Diseases (Excluding Influenza) by Reported Mortality Rates in Mainland China, 2020–2024

Year	AIDS (1/100,000)	Tuberculosis (1/100,000)	Viral Hepatitis (1/100,000)	Rabies (1/100,000)	HFRS (1/100,000)
2020	1.3405	0.1367	0.0419	0.0134	0.0034
2021	1.3919	0.1251	0.0369	0.0106	0.0045
2022	1.3369	0.1561	0.0384	0.0084	0.0024
2023	1.5703	0.1537	0.17	0.0079	0.0009
2024	1.4187	0.1413	0.2291	0.0105	0.0009

Table S21. Reported Cases and Deaths of Monkeypox and Neonatal Tetanus in Mainland China, 2020–2024

Year	Monkeypox		Neonatal tetanus	
	Cases (No.)	Deaths (No.)	Cases (No.)	Deaths (No.)
2020	-	-	34	1
2021	-	-	23	1
2022	-	-	19	1
2023	407	0	21	0
2024	551	0	19	0
Total	958	0	116	3

Table S22. Reported Incidence and Mortality of Notifiable Infectious Diseases in Mainland China, 2016-2019

Year	Cases (No.)	Deaths (No.)	Incidence Rate (1/100,000)	Mortality Rate (1/100,000)
2016	6944063	18234	506.5757	1.3302
2017	7030786	19793	509.5371	1.4345
2018	7770666	23373	559.4026	1.6826
2019	10244442	25280	733.5618	1.8102
Average annual	-	-	577.2693	1.5644
Total	31989957	86680	-	-

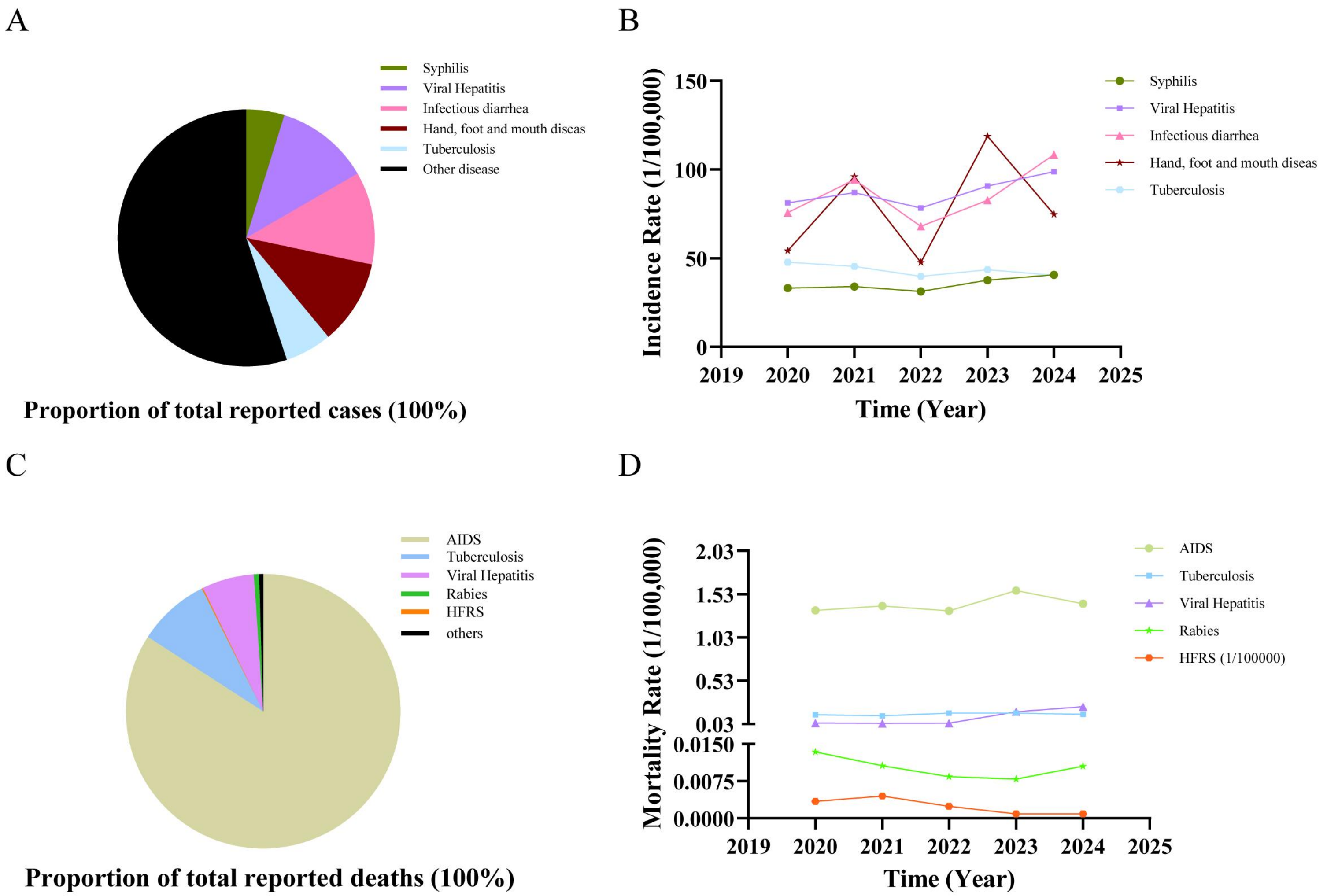

Figure S1. Ranking of Top 5 Notifiable Infectious Diseases (Excluding Influenza) by Reported Cases and Mortality Separately, Mainland China, 2020 – 2024. Proportion of total reported (A)

cases and (C) deaths; (B) Incidence rates of key infectious diseases; (D) Mortality rates of key infectious diseases.
